# Effectiveness of Nature- and Animal Assisted Mindfulness for Relapse Prevention in Depressed Patients With a History of Childhood Maltreatment

**DOI:** 10.3389/fpsyt.2022.899318

**Published:** 2022-07-14

**Authors:** Elisabeth Schramm, Christoph Breuninger, Rainer Wohlfarth, Moritz Elsaesser, Hannah Piosczyk, Thomas Fangmeier

**Affiliations:** ^1^Department of Psychiatry and Psychotherapy, Medical Center and Faculty of Medicine, University of Freiburg, Freiburg, Germany; ^2^Ani.Motion, Institute of Animal Assisted Psychotherapy, Sasbachwalden, Germany

**Keywords:** mindfulness based intervention, nature based, animal assisted, psychotherapy, depression prevention, randomized controlled trial, early life trauma, childhood maltreatment

## Abstract

**Background:**

For relapse prevention in depression, conventional mindfulness programs such as the mindfulness-based cognitive therapy proved to be useful. However, early life trauma is a risk factor for having adverse experiences during meditation. Thus, for this patient group mindfulness skills are often difficult to learn and may be facilitated by using animals and a nature setting.

**Methods:**

The aim of the study was to evaluate the preventative efficacy of a nature- and animal assisted mindfulness program (NAM) over the course of 1 year in unstable or partially remitted depressed patients with a history of early life trauma. NAM included 8 group sessions of 150 min each over 8 weeks plus one booster session. Sixty-seven participants were randomized to either NAM combined with treatment-as-usual (TAU; guideline oriented treatment) or TAU alone. The primary outcome was depression diagnosis over the course of 12 months after end of treatment. Secondary outcomes included clinician- and self-rated depressive symptoms, quality of life, mindfulness skills, and rumination post, and 12 months after the intervention. In addition, we evaluated the participants' satisfaction with the program.

**Results:**

Analyses revealed significant differences in relapse rates and number of weeks depressed throughout the course in favor of NAM. Furthermore, global quality of life improved significantly more in the NAM group. There was no significant difference for other secondary outcomes. Satisfaction with the program was high with a low drop-out rate of 6%. The vast majority of the participants felt safe practicing mindfulness in nature and found sheep for assistance helpful and motivating.

**Conclusions:**

A nature- and animal assisted mindfulness program proved to be feasible, highly acceptable, and more effective than standard treatment in preventing relapses in recurrently depressed patients with childhood maltreatment. Nature and animals can facilitate the engagement in the treatment process for individuals with a history of early trauma. However, further evidence in multicenter trials is necessary.

## Introduction

Relapse prevention is one of the most important goals in depression treatment as major depression is usually a recurrent disorder with a relapsing course. After remission from a depressive episode, continuing (1) or sequentially introducing psychotherapy as add-on to antidepressants (2, 3), can reduce the relapse and recurrence rate in the further course. Of the psychotherapy programs specifically developed for relapse prevention, Mindfulness Based Cognitive Therapy [MBCT; (4)] is effective in remitted patients, particularly in those with frequent depressive episodes (5, 6). However, research and clinical observations raised some concerns with providing mindfulness practices to patients with trauma histories (7) while controversial findings are reported if early trauma could be a risk factor for having adverse experiences during meditation (8). There is evidence that mindfulness practices can be particularly efficacious in preventing depression relapse among people with histories of childhood maltreatment (9, 10), but could be done with more care to reduce risk of adverse events and increase engagement (11–13). While in most meditation studies adverse effects were not systematically assessed, patients with a trauma history often spontaneously report difficulties such as heightened emotional reactions or re-experiencing traumatic memories (7). This kind of passive monitoring of adverse events is thought to underestimate the prevalence of meditation-related negative effects by far (14). Furthermore, there is no consensus on what can be considered an adverse effect in mindfulness trainings. The main challenges with extended sitting practice, body scan or breath awareness for patients with posttraumatic stress disorder (15) or histories of trauma including childhood maltreatment (16) are attributed to over-arousal, distress due to embodied traumatic memories, or feeling overwhelmed with relaxation-induced anxiety and loss of structure. In addition, early maltreated patients reported to feel unsafe lying down with eyes closed in a closed room with other unfamiliar participants (17). Under these conditions, it is difficult to engage in the treatment process and learn mindfulness skills. Therefore, corresponding adaptations and more flexibility in teaching mindfulness have been proposed for this patient group.

Less activating options in a *per se* stabilizing environment such as nature may offer smoother access to painful memories or dysregulative experiences arising from early trauma. Contact with nature has a beneficial effect on mental wellbeing, facilitates positive affect, and reduces anxiety and depressive symptoms (18–20). A recent meta-analytical review suggests that nature-based mindfulness is moderately superior to mindfulness conducted in non-natural settings (21). Moreover, animal-assisted therapy has been shown in several meta-analyses to be effective in reducing stress reactions, depressive and anxiety symptoms, and in increasing motivation (22, 23). There is also evidence for increasing treatment comfort, engagement, and completion by using animals in therapy (24). These findings are underlined by numerous studies indicating that the presence of an animal leads to diminished fear (25–28) and promotes calmness (29, 30).

For teaching mindfulness skills, sheep as flight and social animals proved to be particularly suitable (31) since they show several mindfulness skills themselves. Sheep as intelligent, complex, and feeling individuals sense emotions and moods of human beings and react correspondingly (32). By nature, sheep are non-judgmental and show full awareness in presence. This mirrors the attitude prompted in mindfulness of being present, non-judgmental, accepting, and compassionate which can be especially beneficial for patients with a trauma history. To focus on the here and now facilitates coping with rumination and worries, which are common among maltreated individuals (33). To practice a non-judgmental attitude can be an antidote against feelings of shame, guilt, and anger (34, 35). The cultivation of compassion and particularly of self-compassion (36) decreases self-blame and low self-esteem (37). Animals do not create a therapeutic process *per se*, but they rather support the change processes intended by the therapist (38). In an open pilot study (31), a nature- and animal assisted mindfulness program based on MBCT proved to be feasible and highly accepted by depressed patients. Despite methodological limitations, the results were encouraging and warranted further study with a larger randomized sample.

The aim of the present study was to evaluate the preventative efficacy of the NAM program over the course of 1 year. The primary hypothesis was that participants with a history of depression and childhood trauma randomized to NAM would report significantly less relapses and recurrences during the 1-year assessment period after the intervention. Primary outcome was defined as time to relapse (fulfillment of criteria for Major Depressive Disorder according to DSM-IV-TR) for at least 2 weeks. Secondary outcomes included self-rated [Beck Depression Inventory-II, BDI-II; (39)] and clinician-rated [Hamilton Rating Scale for Depression, HRSD-17; (40)] depressive symptoms, quality of life [WHOQoL; (41)], mindfulness skills [Freiburger Fragebogen zur Achtsamkeit, FFA; (42)], and rumination [Responsiveness Scale Questionnaire, RSQ-D; (43)].

## Materials and Methods

### Trial Design

We conducted a monocentric, randomized controlled trial between July 2016 and October 2019 comparing NAM vs. TAU in a group format in outpatients with unstable or partially remitted depression and a history of childhood maltreatment. Participants were recruited from July 2016 through June 2018, intervention groups were conducted between September 2016 and June 2018, and the last follow-up assessments were taken in September 2019. The Research Ethics Board of the University of Freiburg approved the trial. In accordance with the Declaration of Helsinki, participants were informed in detail about the purpose and design of the trial and provided written informed consent prior to randomization. This trial was registered prospectively with the German Clinical Trials Register (registry number: DRKS00010800).

### Participants and Procedure

**Figure 1:**
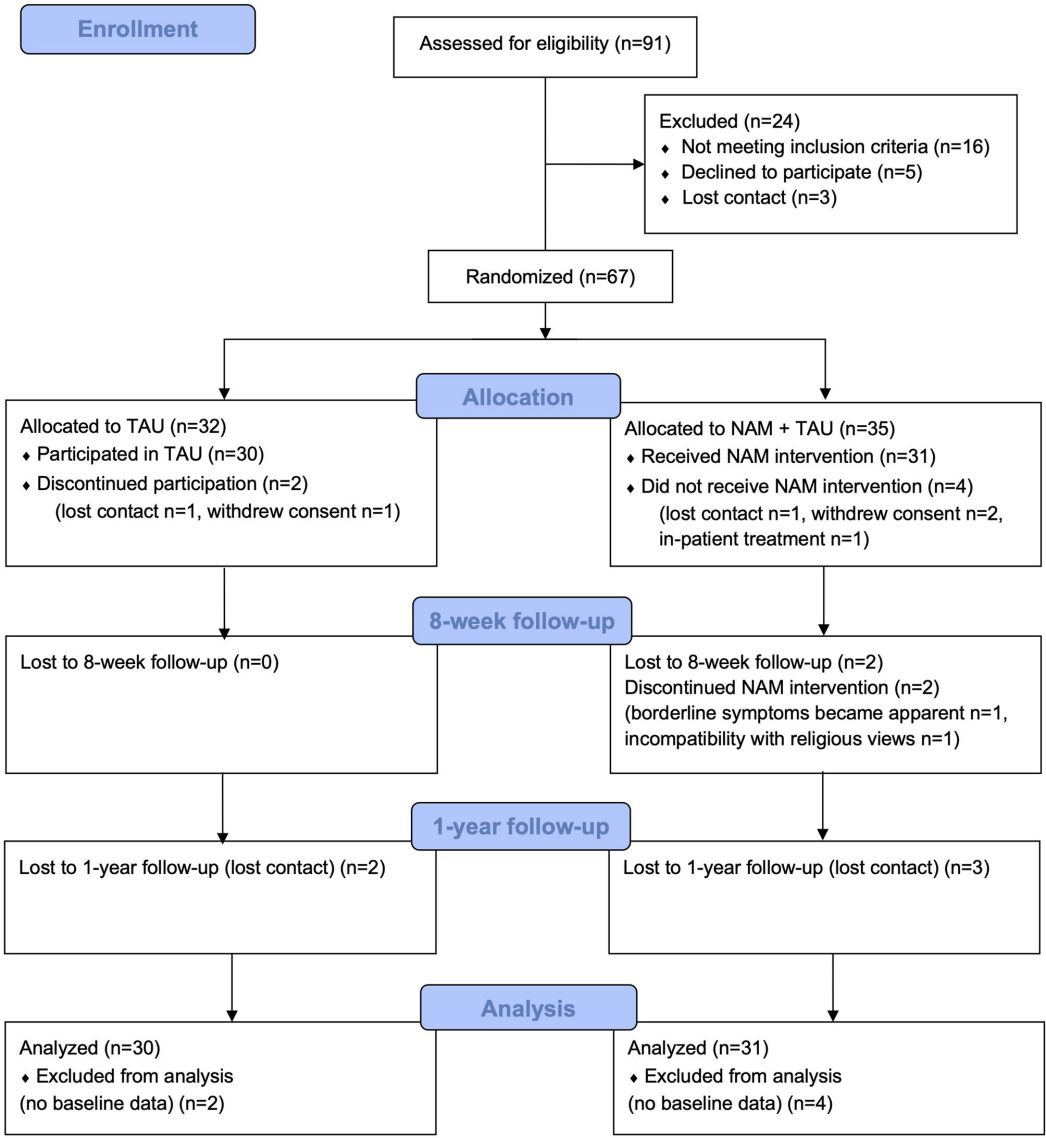
Consort diagram of participant flow.

In total, 67 patients were randomized to either NAM combined with TAU or TAU alone (Figure 1). Randomization was conducted in 5 groups of 10–16 participants, resulting in one NAM and one TAU group each time, according to a central computerized randomization schedule (using random sequences from random.org). Some participants thus had to wait after screening and study inclusion for their randomization group to be complete, which explains the loss of participants prior to baseline assessment and intervention as detailed in Figure 1.

Eligible patients were between 18 and 70 years of age and had a partial or unstable remission of a major depressive episode [i.e., one or more HRSD-17 scores >7 during the 4-week interval before screening, as established by retrospective week-by-week assessment; the criteria of a MDD [Major Depressive Disorder] according to DSM-IV-TR are no longer fulfilled; (44)]. Eligible patients also had at least 3 episodes of a MDD (the last one no longer than 2 years ago) and early trauma experiences according to the self-rated Childhood Trauma Questionnaire [CTQ-SF; (45)] in at least one of the five trauma domains with moderate to severe intensity. All participants had to be in the care of a primary care physician or a psychiatrist throughout the study.

Exclusion criteria comprised a *current* depressive episode, an acute high risk of suicide, a primary diagnosis of another Axis I mental disorder (46); a diagnosis of antisocial, schizotypal, or borderline personality disorder; a serious medical condition; a history of schizophrenia, schizoaffective disorder, bipolar disorder, substance abuse, and organic brain disorders.

Clinical diagnoses were assessed at screening using the Structured Clinical Interview for *DSM-IV-TR* Axis I Disorders [SCID-I; (46)] and the Structured Clinical Interview for *DSM-IV* Axis II Personality Disorders [SCID-II; (47)].

Analogous to the MBCT program (48), the NAM program consists of eight weekly group sessions of 2.5 h duration and one booster session 3 months after termination. The program was conducted with 4–8 participants in a nature setting. NAM is described in a structured guideline (31). The focus was on formal and informal mindfulness and compassion practices in nature and assisted by tamed sheep, psychoeducation on relapse prevention of depressive episodes, and contemplation of practice experiences in the group. The meditation practices included breath awareness, body scan, open awareness, loving-kindness meditation, walking meditation, and mindful eating as well as daily, formal (e.g., body scan), and informal homework (e.g., mindfulness in daily life while walking in nature). An overview of the session contents can be found in the Appendix.

Several modifications to the conventional MBCT program were made to adapt the intervention to our study population (Table 1).

**Table 1:** List of modifications to the conventional MBCT program.

**Modification**	**Explanation**
Interaction with nature	Exercises for mindful observing, describing, and participating are conducted with material from nature (e.g., building stone towers, describing a daisy) and in nature (barefoot walking meditation, eye scan and walking meditation in nature).
	To use natural stimuli for open-awareness meditation, allowing to hold the attention to the present moment more easily.
	The “raisin exercise” is conducted with mindfully self-collected objects from nature (e.g., apples, daisies, cherries, chestnuts, etc.).
	Mindfulness in daily life is based on nature-related everyday tasks (e.g., cleaning enclosures, collecting herbs, walking with sheep in a herd, sheering and wool processing).
	Informal mindfulness is oriented toward the nature setting (i.e., guided attention to the senses like a hearing meditation with an attitude of non-judgment and openness).
Interaction with sheep	Exercises for mindful observing, describing, and participating are conducted with animals (e.g., brushing or leading the sheep)
	Learn to get out of an automatic pilot mode to become aware of the present moment by leading sheep (with immediate feedback from the sheep such as walking in another direction if not present)
	Breathing, compassion, self-compassion, and loving-kindness exercises are conducted with assistance of the sheep.
	In the loving-kindness meditation, the first repeated mantra was sending kind wishes to the sheep or other animals, e.g., “May the sheep be healthy and free of pain.”
Length of meditation exercises	Shortening of the mindfulness meditation exercises from 45 min to 15–20 min, shortening of the body scan exercises to a maximum of 15 min (in the sessions and as homework). Home practice was assigned while instructing the patients to keep a daily log of how many minutes they practiced each skill.

More flexibility on the length of the meditation practices for individuals with a history of childhood maltreatment was also reported in other studies (36, 49). A previous investigation (17) suggested that patients with a trauma history had difficulties practicing over longer periods of time, and found that shorter practices were very helpful for those individuals.

The NAM group program was conducted by a licensed psychotherapist (E. Schramm) with comprehensive training in mindfulness as well as experience with depression treatment and educational work with the sheep participating in the study. The program was accompanied by an animal caretaker from Mundenhof (animal farm run by the city of Freiburg, Germany). Eight tamed “Coburger foxsheep,” a breed of Ovis gmelini aries, participated in the program. They are familiar with interacting with human beings and trained to walk with or without a leash. The animals' stress level during the meetings was continuously monitored by the animal caretaker joining the program. No signs of stress (e.g., restlessness, frequent urination, accelerated breathing, etc.) were detected in the animals. On the contrary, they actively sought contact with the participants. All training sessions took place outside at the Mundenhof farm which is surrounded by forests and meadows.

Patients randomized to TAU were encouraged to continue with guideline-oriented treatment (including psychotherapy and/or pharmacotherapy by a primary care physician, psychiatrist, or licensed psychotherapist) throughout the study period. In order to compensate for participation in the study assessments, patients in the TAU condition were offered to participate in the NAM program after their follow-up evaluation.

### Assessments

The following assessments were taken pre-treatment, post-treatment and at one-year follow-up (with parallel assessments in the TAU group), with the exception of the Beck Depression Inventory-Fast Screening [BDI-FS (50)]. All clinician ratings were conducted by blinded, trained, and experienced raters. The primary outcome was the course of depression 12 months after termination of the NAM program measured with a stepped diagnostic process of monthly BDI-FS and subsequent phone interviews.

BDI-FS (50): The screening of self-assessed depressive symptoms with the BDI-FS consists of seven items out of the BDI-II. This screening tool is designed to keep the repeated assessments of depressive symptoms as short as possible. The calculated transformation of the total score of the BDI-FS into an equivalent BDI-II score is described in the BDI-FS manual (50) (p. 11). In different studies, a high correlation between both instruments could be found (*r* = 0.85 to *r* = 0.92) (51, 52). Through a combination of a number of specific items and a minimum total score, sufficiently high sensitivity and specificity is reached. In an American sample, major depression was correctly classified with a sensitivity of 87% and a specificity of 85% with this algorithm (53). Participants were prompted monthly (and if necessary, reminded) by e-mail and completed the BDI-FS form online.

SCID & HRSD-17 telephone interview: participants with a sum score of at least five points (and at least one point on either the depressed mood or anhedonia items) were contacted by telephone, and a HRSD-17 (40) rating and SCID-I (46) assessment (affective disorders section) were performed.

Beck Depression Inventory-II [BDI-II; (39)]: The self-rated questionnaire includes 21 items to assess depressive symptoms from the patient's perspective. The test quality criteria, such as internal consistency, validity, and test-retest reliability, are most satisfactory in both clinical and non-clinical subjects (Cronbach's alpha = 0.89).

Hamilton Rating Scale for Depression (HRSD-17): The HRSD-17 is a clinician administered clinical interview for the assessment of the severity of depressive symptoms. Scores range from 0 to 51, with higher scores reflecting more depressive symptoms. A score of 0–8 is qualified as “normal,” 9–16 as “mild,” 17–24 as “moderate,” and a score ≥ 25 as “severe.” Detailed information on psychometric properties of the HRSD-17 can be found elsewhere (54).

Freiburger Fragebogen zur Achtsamkeit [FFA; (42)] is a one-dimensional self-rated questionnaire with 14 items for the evaluation of different aspects of mindfulness and associated skills. The measure has satisfactory test quality criteria.

Responsiveness Scale Questionnaire [RSQ-D German version; (43)] includes 32 items distributed on two dimensions: rumination and distraction. The measure has satisfactory test quality criteria.

Quality of Life [WHOQoL-Bref; (41)] contains 26 items related to 4 different dimensions: The physical domain, the mental domain, social relationships, the environment domain. The global domain includes items to assess life quality and health satisfaction. The measure has satisfactory test quality criteria.

In addition, early trauma experiences were assessed at baseline with the short form of the Childhood Trauma Questionnaire [CTQ-SF; (45)]. The CTQ is a 28-item retrospective self-report questionnaire evaluating the dimensions emotional abuse, physical abuse, sexual abuse, emotional neglect, and physical neglect that occurred during childhood and/or adolescence (“when they were growing up”). Exposure frequencies using a five-point Likert scale were summed up for domain scores. Cutoff scores for each domain were used to assign categories of “none to minimal,” “low to moderate,” “moderate to severe,” and “severe to extreme.” The German version of the CTQ has been shown to be a reliable and valid instrument (55).

To assess the participants' satisfaction with the NAM intervention as well as their experiences with practicing mindfulness in nature and with animal assistance, we used a self-designed questionnaire with 23 items. Of these, 18 items (5- point-scale ranging from 1 = “fully disagree” to 5 = “fully agree”) were averaged into three scales: *nature helpful* (6 items, e.g., “The mindfulness exercises in nature were easy for me to carry through.”, “The practice setting in nature allowed me to get involved in the mindfulness exercises.”), *animals helpful* (10 items, e.g., “The presence of the animals allowed me to get involved in the mindfulness exercises.”, “The ‘mindfulness' of the animals served as a model for me.”, “I found sheep suitable animals for the mindfulness program.”), and *flooded/agitated* (2 items: “I was flooded with images, feelings, or early memories during mindfulness practice.”, “At times I was agitated by the mindfulness practice.”).

### Sample Size

The sample size calculation was based on the relapse rates in a meta-analysis of MBCT for relapse prevention in MDD (5), specifically in the subgroup with 3 or more prior depressive episodes, reported as 63% for TAU and 35% for MBCT. Assuming this reported relapse rate in our TAU group and a somewhat improved relapse rate of 31% in the NAM group resulted in a sample size of 64 patients for the primary survival analysis (with a power of 80% and the conventional alpha error of 5%), calculated with Stata 13.1.

### Statistical Methods

The primary efficacy analysis was performed according to the intention-to-treat (ITT) principle and therefore was based on the full analysis set (FAS), assuming data missing at random. The FAS included all randomized patients, and patients were analyzed according to their randomized arm regardless of protocol deviations. Patients dropping out before the baseline assessment were excluded (compare Figure 1). Statistical analyses were performed in R version 4.1.0 (56).

The primary endpoint (time to first relapse of MDD during the course of 12 months after termination of the NAM program, as determined by SCID-I by telephone if monthly BDI-FS ratings indicated elevated depressive symptoms) was analyzed via log-rank test of the two survival curves (NAM vs. TAU) using the R survival package (57).

**Figure 2:**
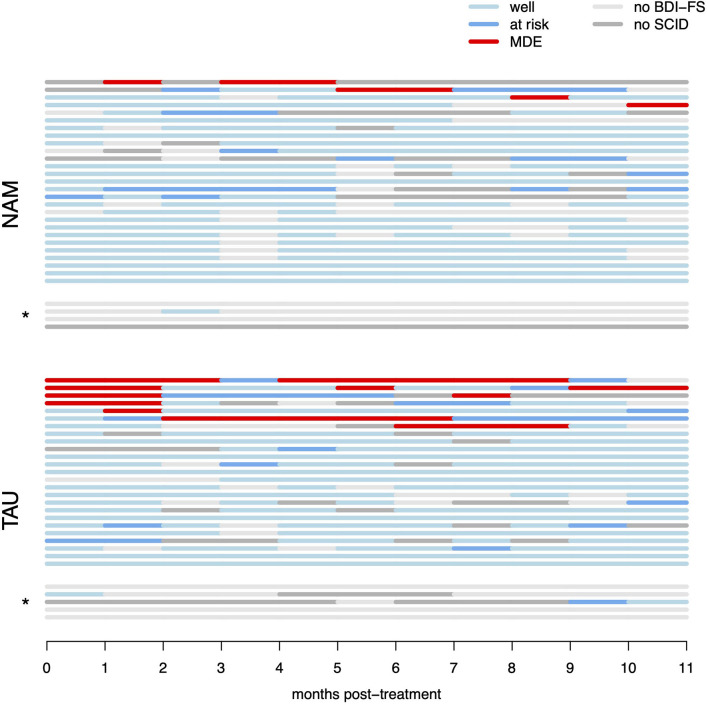
Raw survival data (monthly per-participant data regarding recurrence of MDD). “Well” denotes months with sub-threshold BDI-FS scores (and no diagnostic interview performed), “at risk” denotes months with elevated BDI-FS scores, but criteria for MDE were not met in the diagnostic interview, “MDE” denotes months with elevated BDI-FS scores and diagnosis of major depressive episode in the diagnostic interview by telephone. “No BDI-FS” denotes months for which BDI-FS data could not be obtained, “no SCID” denotes months with elevated BDI-FS scores, but participants could not be reached for the diagnostic interview. *indicates participants with <3 months of available monthly data, which were excluded from the multiple imputation analysis (but included in the raw data ITT survival analysis).

Missing data (and thus censoring of survival data) was an issue in the monthly BDI-FS and SCID data (compare Figure 2), with a mean (SD) of 3.87 (3.50) months of missing data in NAM and 3.33 (3.61) in TAU group. Thus, after performing the pre-specified ITT analysis on the raw data, we imputed the MDD status for the missing months using the R Amelia package (58). Only cases with at least 3 of the 11 monthly measurements were used (excluding *n* = 4/5 cases from NAM/TAU groups, respectively, and leaving *n* = 27/25 for the imputed analysis). The available monthly measurement data was included in the imputation model as lags and leads (thus preserving the information on the sequential order), as well as monthly BDI-FS status when only the depression diagnosis was missing because the SCID-I interview couldn't be conducted in time. The model further included baseline CTQ and baseline, post and follow-up BDI-II, HRSD-17, and WHOQoL global and mental domains. The resulting continuous predictions of MDD status were dichotomized such that the percentage of MDDs in observed and missing months was similar (~10%). Imputation diagnostics by over-imputation and visual inspection of predicted values in the timelines indicated successful prediction. The primary analysis was repeated on 100 imputed datasets, combining the resulting Chi-Square values and testing for significance with the R miceadds package (59). Missing items in questionnaire data were rare (<1%) and were imputed with iterative robust model-based imputation from the VIM R package (60) for observations with <20% missing items. Visual inspection of raw data and model residuals indicated that no outliers were present in the data.

Secondary outcomes were analyzed as linear mixed models with R package lme4 (61), with fixed effects for time (pre-treatment, post-treatment and 1 year follow-up) and group (NAM vs. TAU) as well as their interaction effect, using a random intercept for each participant to account for the repeated measurements. The main test of interest was the chi-squared test of the interaction effect. Further, contrasts of interest were tested using R package lmerTest (62). Treatment contrasts of the interaction between time and group were performed with pre-treatment values as baseline, thus testing for group differences changes from pre- to post-treatment as well as pre-treatment to follow-up.

### Sample

Participants were predominantly female, middle aged, and with rather high educational status (Table 2). Only a minority worked full-time, with high rates of marginal employment and unemployment.

**Table 2:** Participant characteristics.

	**NAM**	**TAU**	**Overall**
	**(*N* = 31)**	**(*N* = 30)**	**(*N* = 61)**
**Gender**			
Female	25 (80.6%)	24 (80.0%)	49 (80.3%)
Male	6 (19.4%)	6 (20.0%)	12 (19.7%)
**Age (years)**			
Mean (SD)	47.5 (13.2)	48.7 (12.9)	48.1 (13.0)
**Persons living in household**			
1	19 (61.3%)	13 (43.3%)	32 (52.5%)
2	7 (22.6%)	9 (30.0%)	16 (26.2%)
3 or more	4 (12.9%)	8 (26.7%)	12 (19.7%)
**Schooling**			
lower secondary (9–10 yrs)	14 (45.2%)	9 (30.0%)	23 (37.7%)
higher secondary (12–13 yrs)	17 (54.8%)	21 (70.0%)	38 (62.3%)
**Current occupation**			
full time	5 (16.1%)	10 (33.3%)	15 (24.6%)
part time	11 (35.5%)	8 (26.7%)	19 (31.1%)
Other	4 (12.9%)	4 (13.3%)	8 (13.1%)
not employed	11 (35.5%)	8 (26.7%)	19 (31.1%)
**HRSD-17 score at screening**			
Mean (SD)	10.7 (4.00)	11.6 (4.85)	11.1 (4.42)
**HRSD-17 variation at screening**			
Mean (SD)	3.52 (2.79)	3.80 (3.59)	3.66 (3.18)
**Prior experience with mindfulness**			
Yes	16 (51.6%)	17 (56.7%)	33 (54.1%)
No	14 (45.2%)	13 (43.3%)	27 (44.3%)
**Ever lived with a pet**			
Yes	29 (93.5%)	25 (83.3%)	54 (88.5%)
No	2 (6.5%)	4 (13.3%)	6 (9.8%)
**Nature-loving (0–100)**			
Mean (SD)	93.7 (9.74)	89.2 (16.0)	91.5 (13.3)
**Animal-loving (0–100)**			
Mean (SD)	91.3 (16.8)	86.6 (21.0)	89.0 (19.0)

*Note: HRSD-17 score at screening reports the highest score from the four weekly values obtained during screening, HRSD-17 variation reports the difference between the highest and lowest of these measures*.

Due to difficulties in recruiting enough participants for timely randomization into the required groups, 19 participants with elevated, yet less than moderate CTQ scores and early onset of depression were included. Similarly, 13 participants with HRSD-17 scores of 7 or lower during screening were included, when the clinical judgement indicated partial or instable remission. The degree of childhood maltreatment ranged from having suffered mild abuse in only few dimensions, to extreme abuse and neglect in several dimensions (Table 3). Most common were emotional abuse and neglect by about three quarters of the participants. In both groups, about 30% reported no dimensions with moderate or higher severity, about 25% reported 1 dimension, and 45% reported 2–5 dimensions. HRSD-17 scores were in the subthreshold range as expected, with considerable variation even between the four weekly assessments obtained during screening. Only half of participants had prior experience with mindfulness exercises, mostly from in-patient settings.

**Table 3:** Dimensions and severity of childhood traumatization (CTQ) by group.

**Severity**	**Emotional abuse**		**Physical abuse**		**Sexual abuse**		**Emotional neglect**		**Physical neglect**	
	**NAM**	**TAU**	**NAM**	**TAU**	**NAM**	**TAU**	**NAM**	**TAU**	**NAM**	**TAU**
None to minimal	23% (7)	37% (11)	71% (22)	87% (26)	65% (20)	63% (19)	16% (5)	17% (5)	55% (17)	60% (18)
Low to moderate	23% (7)	27% (8)	6% (2)	3% (1)	16% (5)	13% (4)	29% (9)	37% (11)	16% (5)	13% (4)
Moderate to severe	29% (9)	17% (5)	13% (4)	10% (3)	10% (3)	3% (1)	16% (5)	20% (6)	19% (6)	17% (5)
Severe to extreme	26% (8)	20% (6)	10% (3)	0% (0)	10% (3)	20% (6)	39% (12)	27% (8)	10% (3)	10% (3)

*Note: Percentages of participants within group and dimension (i.e., column) are presented, and the absolute number (in brackets). N = 31 for NAM and N = 30 for TAU group*.

## Results

### Primary Outcome

The test for the primary hypothesis indicated a significant difference between the survival curves in NAM vs. TAU groups [$\chi_{\left(1\right)}^{2}$ = 6.62, *p* = 0.010]. This suggests, that participants in the NAM group experienced fewer relapses and fewer weeks in MDD compared to the TAU group. However, this result has to be interpreted cautiously because of a high level of censoring resulting from missing measurements in the monthly assessments (see Statistical Methods above). All four of the MDD relapses observed in the NAM group (13% relapse, n_NAM_ = 31) are not captured in the raw survival model because of missing data occurring first (i.e., censoring). In contrast, only one of the seven MDD relapses observed in the TAU group (23% relapse, n_TAU_ = 30) is missed (censored) for the same reason. This is partly due to MDD relapses occurring later in the NAM group as hypothesized (with four of the TAU relapses occurring in the first month of observation already), but nonetheless this reduces the robustness of the raw survival analysis (compare Figure 2). The repeated primary analysis on 100 imputed datasets failed to reach statistical significance [*F*_(1, 2496.21)_ = 0.915, *p* = 0.339].

### Secondary Outcomes

The number of observed months in depression added up across all participants was 7 months in the NAM and 27 months in the TAU group in the raw data, with an individual mean time in depression of 0.23 months (SD = 0.67) per NUM participant and 0.90 months (SD = 1.97) per TAU participant. However, the between-groups *t*-test was on the border to statistical significance [*t*_(35.4)_ = 1.77, *p* = 0.084]. Repeating the between-groups test with the multiply imputed dataset and pooling with the R package Zelig (63) also failed to reach statistical significance (*z* = 1.41, *p* = 0.158). In the imputed datasets, each patient in NAM group was in depression for 0.54 months on average, patients in TAU group for 1.28 months.

**Figure 3:**
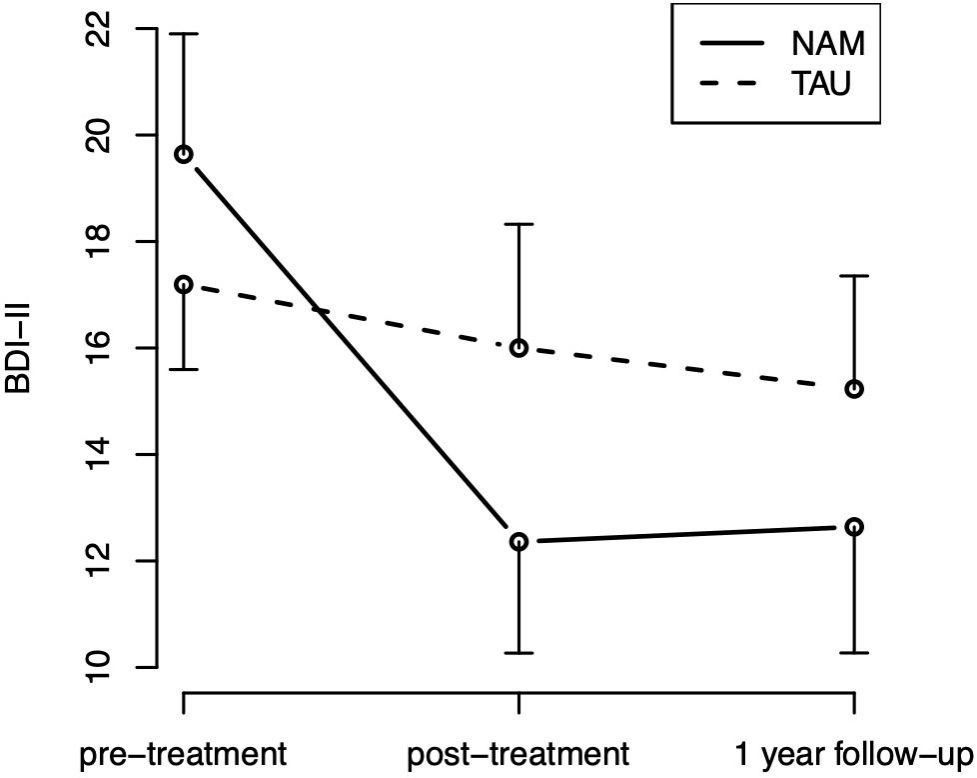
BDI-II time course by treatment group (means and standard errors).

For the HRSD-17, neither the effect of time [$\chi_{\left(2\right)}^{2}$ = 2.52, *p* = 0.284] nor treatment group [$\chi_{\left(1\right)}^{2}$ = 0.16, *p* = 0.692), nor the hypothesized group-by-time interaction ($\chi_{\left(2\right)}^{2}$ = 0.73, *p* = 0.696] were statistically significant. For the BDI-II, the effect of time was statistically significant [$\chi_{\left(2\right)}^{2}$ = 11.39, *p* = 0.003], indicating an improvement over time in self-rated depressive symptoms, which appears especially pronounced in the NAM group (Figure 3). But neither the main effect of group [$\chi_{\left(1\right)}^{2}$ = 0.22, *p* = 0.642] nor the hypothesized group-by-time interaction [$\chi_{\left(2\right)}^{2}$ = 3.39, *p* = 0.183] were statistically significant.

**Figure 4:**
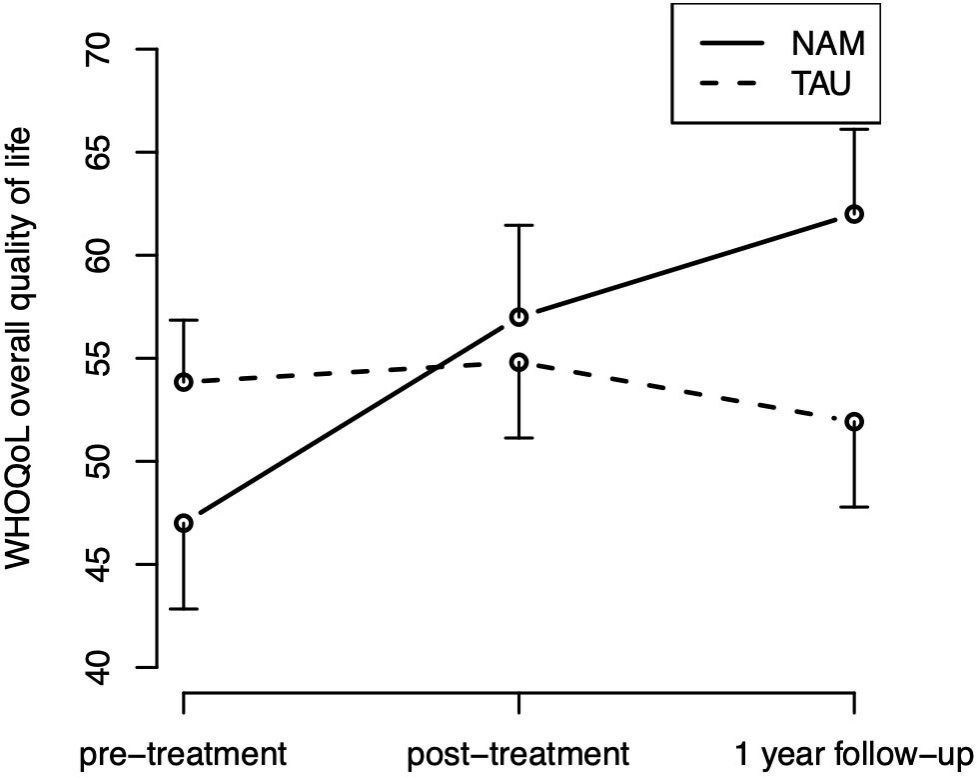
Overall quality of life (WHOQOL) time course by treatment group (means and standard errors).

For the overall quality of life (WHOQOL-BREF), the hypothesized group-by-time interaction [$\chi_{\left(2\right)}^{2}$ = 7.08, *p* = 0.029] was statistically significant, The corresponding contrasts indicate that the group difference in improvement from pre- to post-treatment was not statistically significant [*t*_(107.9)_ = 0.35; *p* = 0.727], but the improvement from pre-treatment to follow-up was [*t*_(109.9)_ = 2.42; *p* = 0.017], indicating an improvement over time in quality of life in the NAM group particularly in the long run (Figure 4). Neither the main effect of time [$\chi_{\left(2\right)}^{2}$ = 5.03, *p* = 0.081] nor group [$\chi_{\left(1\right)}^{2}$ = 0.53, *p* = 0.465] were statistically significant. The hypothesized group-by-time interaction did not reach statistical significance for any of the other secondary outcomes [WHOQOL subscales, mindfulness skills (FFA), and rumination (RSQ), *p*s > 0.15].

### Participants' Program Evaluation

Twenty-four (of 29) participants filled in an evaluation form after completing the program. Overall satisfaction was high, with 18 (75%) reporting being very satisfied with the program, 5 (21%) being satisfied, and 1 person (4%) neutral (on a 5-point scale from very satisfied to very unsatisfied). A majority of 18 participants (75%) experienced the duration with 8 weekly sessions as too short, the remaining 6 (25%) rating the duration as just right (on a 3-point scale from too long to too short).

**Figure 5:**
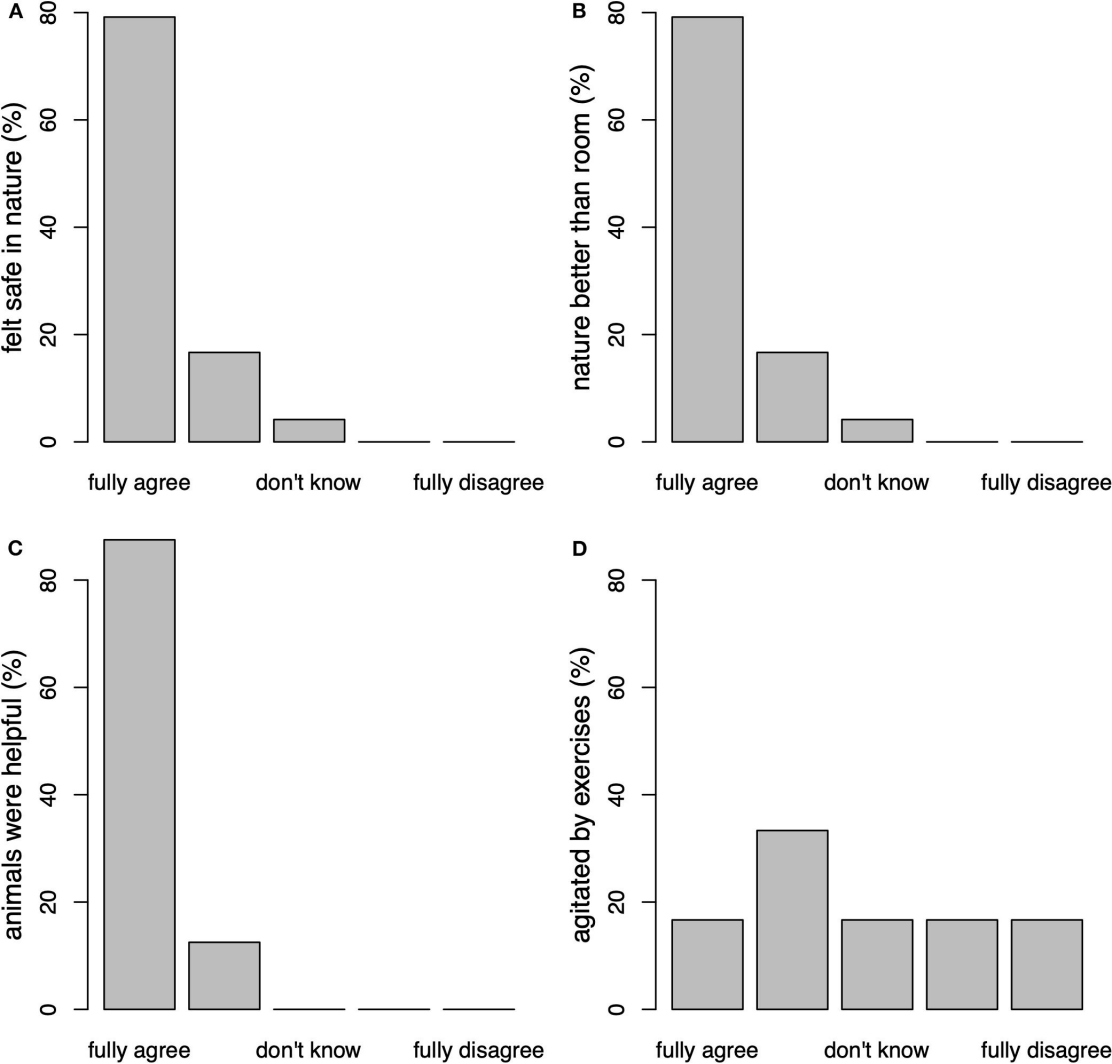
Participants experiences with the NAM intervention (percentages of responses on each 5-point scale, total *n* = 24 for all scales). **(A)** “I felt safe and grounded because of the natural environment” **(B)** “I prefer a therapy program in nature rather than clinic rooms” **(C)** “Contact with the animals was helpful for me” **(D)** “At times I was agitated by the mindfulness practice.”

Participants rated nature as very helpful, with a mean (SD) score of 4.72 (0.33) on the scale from 1 to 5. Similarly, the involvement of animals was rated as very helpful with a mean score of 4.67 (0.37). The amount of agitation experienced during exercises was rated as moderate, with a mean score of 3.08 (1.09). Figure 5 displays the responses to individual items of interest from these scales.

## Discussion

The aim of the present study was to evaluate the preventative efficacy of a nature- and animal assisted mindfulness program over the course of 1 year in high-risk patients (i.e., unstable or partially remitted with a history of early life trauma and three or more depressive episodes). There was a significant difference between the survival curves in NAM vs. standard treatment. The results suggest fewer relapses and months in depression in the NAM group. While relapses were relatively rare in our sample overall, both the number of participants with relapses as well as the individual duration and number of relapses appear reduced in the NAM group (compare Figure 2), resulting in only about a quarter of observed months in depression compared to TAU. Self-rated depressive symptoms and rumination decreased, and mindfulness skills increased in the course of the program in both groups with no significant difference. The global quality of life improved significantly more in the NAM group.

Another goal of this study was to determine the feasibility and acceptance of the program by the participants. Compared to attrition rates of 29.2% (64) in other studies using mindfulness approaches, we observed a low dropout rate of only 6% in the NAM group suggesting high compliance with the intervention among the patients. However, the specific retention rate among patients with childhood maltreatment is largely unknown (8), but expected to be higher than in patients without childhood maltreatment. In addition, the satisfaction with the NAM program was very high: The vast majority of participants in the intervention group were “very satisfied” or “satisfied” with the program, although wishing for a longer duration of the intervention. Most patients felt safe and grounded practicing mindfulness in nature. While almost all patients fully agreed that sheep were helpful and motivating as assistance throughout the sessions, only a few (17%) still felt sometimes agitated during the exercises. The use of nature and animals facilitated the engagement in the treatment process and prevented participants from dropping out. This might have also been influenced by the fact that almost all participants rated themselves as animal and nature lovers, a question that is often discussed as a limitation in the literature, however rarely assessed.

Since increasing mindfulness skills did not seem to be the main driver of effects, we assume as potential mechanisms of NAM that the assistance of nature and animals led to a decrease of arousal and enabled the patients to feel safe enough to get fully involved in the program. Thus, by increasing treatment engagement, the learning of general coping skills was facilitated and in this way might have reduced depression symptoms and relapses in the long term.

Our results are in line with a recent meta-analytical review suggesting that nature-based mindfulness shows advantages compared to mindfulness programs in non-natural settings (21). Mindfulness in wild nature—as practiced in our study—seems to be more beneficial than mindfulness in more cultivated settings such as parks or gardens, but the importance of the setting needs further investigation. To our knowledge, this is the first study using a nature- as well as an animal assisted setting in a mindfulness program. There is very limited prior empirical research on the clinical effects of conventional mindfulness-based interventions in adult individuals with childhood maltreatment (8). The wide variety of animals used, the duration of the intervention, outcome measures, control conditions, and methodological quality make it difficult to compare across studies utilizing animal assisted therapy. None of the studies used monthly assessments of relapses which is one of the strengths of our trial. It is criticized in the literature that despite hundreds of published studies on this issue, there is only limited evidence for the efficacy of the incorporation of animals in the treatment of mental disorders as these studies frequently suffer from lack of methodological rigor. Even though in recent years the quality of animal assisted therapy research has gotten better, most studies still use very small sample sizes, no appropriate control groups, no blinding of raters, no assessments of long-term effectiveness, no manualization of animal interventions, selective reporting of outcome measures or other weaknesses in the research designs (65–67). In our study, most of those quality characteristics were considered.

There are also several limitations of the present study. First, patients were heterogeneous in terms of baseline severity of depressive symptoms and severity of childhood trauma. Aggravating this issue, we had to allow for an extended use of inclusion criteria (severity of HRSD score and CTQ-score) for recruitment reasons. Furthermore, it proved difficult to obtain monthly assessments from patients in this chronically impaired clinical group who had to respond both to monthly e-mail invitations for an online questionnaire and be contacted by telephone in case of elevated values. The multiple imputation of missing values provides important context, but plausibly made it harder to detect meaningful differences in combination with the primary survival analysis, because the purposeful introduction of random variation in multiple imputation runs counter to the high importance the survival analysis places on singular events. In combination with a relapse rate generally lower than anticipated, this probably led to a dilution of treatment effects in the multiple imputation procedure through the introduction of “random” relapses. At the same time, the analysis of the raw data is affected by the survival analysis' sensitivity to missing data, resulting in the loss of information after missing months (i.e., “censoring”). As noted in the methodological literature, a perspective of clinical improvement and mental health as continuous variables might be more helpful (68, 69).

In conclusion, in a study environment including mainly individuals who like animals and nature, a nature- and animal assisted mindfulness program for relapse prevention proved to be feasible and highly acceptable among patients with trauma histories. NAM turned out to be more effective than standard treatment in preventing relapses and weeks in MDD in our study population. Further evidence should routinely include measurement of early trauma to increase the amount of empirical data on this topic. We found that nature and animals can facilitate the engagement in the treatment process for individuals with a history of childhood maltreatment. However, more research is needed on trauma sensitive mindfulness-based protocols including nature- and animal assistance. Since this form of nature- and animal-assisted mindfulness program was never investigated before, the first step was to compare the approach with treatment-as-usual. Future work could directly compare the effects to conventional MBCT programs among patients with and without childhood maltreatment histories.

## Data Availability Statement

The raw data supporting the conclusions of this article will be made available by the authors, without undue reservation.

## Ethics Statement

The studies involving human participants were reviewed and approved by Ethics Committee of the University of Freiburg. The patients/participants provided their written informed consent to participate in this study.

## Author Contributions

ES and CB had full access to all the data in the study and take responsibility for the integrity of the data and the accuracy of the data analysis. ES, TF, and RW: study concept and design. ES, CB, TF, HP, and ME: acquisition, analysis, or interpretation of data and drafting of the manuscript. CB: statistical analysis. ES: obtained funding and administrative, technical, or material support and study supervision. All authors: critical revision of the manuscript for important intellectual content. All authors contributed to the article and approved the submitted version.

## Funding

This study was funded by the Stiftung zur Förderung der Erforschung von Zivilisationserkrankungen, Baden-Baden, Germany. The funders had no role in study design, data collection and analysis, or preparation of the manuscript.

## Conflict of Interest

The authors declare that the research was conducted in the absence of any commercial or financial relationships that could be construed as a potential conflict of interest.

## Publisher's Note

All claims expressed in this article are solely those of the authors and do not necessarily represent those of their affiliated organizations, or those of the publisher, the editors and the reviewers. Any product that may be evaluated in this article, or claim that may be made by its manufacturer, is not guaranteed or endorsed by the publisher.
